# Thermoforming Characteristics of PLA/TPU Multi-Material Specimens Fabricated with Fused Deposition Modelling under Different Temperatures

**DOI:** 10.3390/polym14204304

**Published:** 2022-10-13

**Authors:** Neilson Peter Sorimpuk, Wai Heng Choong, Bih-Lii Chua

**Affiliations:** 1 Department of Mechanical Engineering, Politeknik Kota Kinabalu, Kota Kinabalu 88450, Malaysia; 2 Faculty of Engineering, Universiti Malaysia Sabah, Kota Kinabalu 88400, Malaysia

**Keywords:** fused deposition modelling, multi-material, thermoforming, thermoforming temperature

## Abstract

Multi-material products are required in fused deposition modelling (FDM) to meet a desired specification such as a rigid structure with soft material for impact protection. This paper focuses on the thermoformability and shape recovery characteristics of three-dimensional (3D)-printed multi-material specimens under different thermoforming temperatures. The multi-material specimens consist of polylactic acid (PLA) and thermoplastic polyurethane (TPU). The PLA/TPU specimens were prepared by depositing the TPU component on top of the PLA component using a fused deposition modelling (FDM) machine. Simple thermoforming tests were proposed, where the specimens were bent under load and molded into a circular shape at different thermoforming temperatures. The bent specimens were then reheated at 60 °C to evaluate their shape memory ability. The test results were quantified into apparent bending modulus and shape recovery percentage. The PLA/TPU specimens showed a better apparent bending modulus of 143 MPa than a PLA specimen at a temperature between 60 °C to 90 °C. However, only the PLA/TPU specimens being thermoformed into a circular shape at 100 °C or greater showed good shape retention accuracy and interfacial surface bonding. The PLA/TPU specimens that were thermoformed at 60 °C to 90 °C showed reasonable shape memory of about 60% recovery when reheated. Finally, suitable thermoforming temperatures for thermoforming PLA/TPU specimens were suggested based on design needs.

## 1. Introduction

Fused deposition modelling (FDM) is commonly used for rapid prototyping. This method is based on the extrusion of molten filament as the material which is being dispensed layer by layer through a heated nozzle according to a sliced model [1,2]. Although the product produced by this method is rigid and accurate, the deposition process time is long to create a three-dimensional (3D) object [3]. In order to minimize the time consumption for creating a 3D object using the FDM process, various techniques have been studied, including creation of 3D objects via post-deposition shape modification of a flattened object via the thermoforming method [4].

In the FDM-thermoforming method, the intended model is printed as a flat-shaped object and reshaped into the desired product with the application of heat and pressure [5]. The most common material used for the FDM-thermoforming process is polylactic acid plastic (PLA). This is due to its material properties that allow it to be easily deformed at low temperatures [6]. PLA is made from recyclable natural raw materials and is known for its biodegradability and biocompatibility to the human body [7]. As a thermoplastic, it can be reshaped when heated several times without losing its mechanical properties. This thermoforming method has been experimented on to produce a complicated object without consuming much fabrication time, such as prosthetic hands with various sizes and shapes [8,9,10]. It greatly improved the accuracy and fabrication time of the final product to fit directly to the necessary shape. In a recent study by Choi et al., they concluded that a 3D-printed hand splint, which had undergone thermoforming, fit more accurately to the user’s hand as compared with those printed directly based on 3D scanning data due to muscle movement during the scanning process [11]. The splint, made of PLA, is rigid and may result in discomfort when being worn for a long duration. In order to improve the comfort and functionality of the 3D-printed and thermoformed products, utilization of soft material such as thermoplastic polyurethane (TPU) combined onto the rigid PLA material is desired. TPU is a flexible and elastic type of material that possesses rubber-like elasticity, resilience, and durability. It is desirable in the applications that require impact absorption and a soft-touch surface. The adhesion of the PLA/TPU material should be maintained after the thermoforming process to render the usefulness of the printed product. For this purpose, this paper focuses on direct printing of different materials into a layered structure, although the PLA/TPU multi-material polymers via FDM can also be formed through two other methods: utilization of pre-mixed filament blends of polymers with different proportions, and application of pre-mixed composite filaments consisting of a polymer matrix with the inclusion of filler [12,13,14,15,16].

The challenge of FDM direct-printed multi-material parts is their adhesion. Several researchers investigated the printing parameters on adhesion and properties of multi-material 3D printing for the multi-material structure made with a combination of PLA and TPU [13,17,18]. Brancewicz-Steinmetz et al. found that proper printing parameters and TPU on PLA print sequences result in better adhesion between materials [17]. Kepenekci and Zhang proposed implementation of a mechanical lock mechanism and bio-inspired arrangement of the structure to improve the adhesion between the printed PLA/TPU interface, respectively [18,19]. The combination of rigid PLA and soft TPU material provides load absorption while limiting cracks [17]. These studies supported the possibility of combining PLA/TPU materials via the FDM process to achieve a desirable mechanical characteristic of prints. Studies that investigated bonding in multi-material additive manufacturing are mainly based on tensile tests [15,19]. However, the characteristics of the PLA/TPU structure fabricated using the additive process of FDM after a thermoforming post-process are not well understood, and thus hinders its potential in additive manufacturing.

On the other hand, Dogan et al. and Razzaq et al. reported that the PLA/TPU polymer has shown a shape memory behavior [12,16]. Dogan et al. investigated the shape memory properties of the printed specimen by tensile tests [16]. An et al. and Kačergis et al. reported that the printing speed of PLA has considerable influence, as the structures printed with higher speeds bend more during the shape memory activation by using heat, and a significant increase can be seen at the 80 mm/s print speed [20,21]. While the layer thickness contributes to the printing time consumption, there is no clear effect of the layer thickness on mechanical properties of PLA specimens [22].

Material selection for thermoforming is carried out based on basic mechanical tests such the flexural stiffness test ASTM D790, tensile strength test ASTM D638, and heat deflection temperature test ASTM D648 [23]. However, there is no known standard that directly tests the thermoformability and shape memory characteristics of a multi-material plastic. For more accurate data, product testing on a finished and fully assembled specimen are usually conducted. Test setups in the literature were set based on the characteristics of the product of their interest. This has been evidenced by different test methods by various researchers [4,24,25,26,27]. Ekşi investigated the thermoforming of a 3D-printed part by evaluating the shape accuracy of the product being thermoformed into a mold [24]. Mustakangas et al. studied the thermoformability of 3D-printed PLA specimens by pressing them into an arch shape under a fixed temperature of 100 °C [4]. These methods were limited to a single-material product and are not suitable to evaluate the adhesion between the multi-material part. Wang et al. prepared laminate structures of PLA/TPU and evaluated their shape memory performance using the unfolding angle of a bent specimen [27]. Li et al. proposed recovery experiments derived from a three-point bending test to study the bending shape recovery of a shape memory polymer composite at a fixed temperature [28]. The effect of thermoforming temperature on shape memory was not investigated. Simple thermoforming and shape memory tests for the thermoformed multi-material structure using an inexpensive apparatus are necessary to enable more researchers and 3D printing hobbyists to easily assess and compare their products.

This paper focuses on the thermoformability and shape recovery characteristics of three-dimensional (3D)-printed PLA/TPU specimens under different thermoforming temperatures. Three simple experiments are introduced. Bending and circular shape molding tests are performed at different thermoforming temperatures. The shape memory ability is tested by reheating the deformed specimens. Finally, suitable temperatures for thermoforming PLA/TPU specimens are suggested to obtain high thermoformed shape accuracy and to avoid layer delamination.

## 2. Preparation of PLA/TPU Specimen

**Figure 1:**
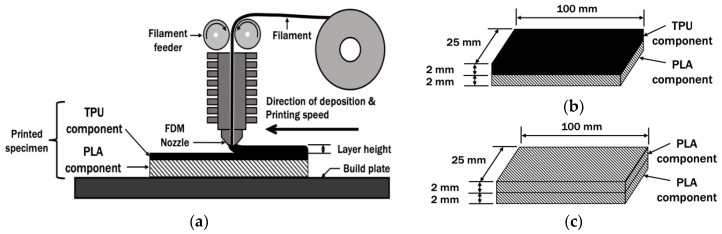
Specimen models. (**a**) FDM process of specimen; (**b**) dimension of PLA/TPU specimen; (**c**) dimension of PLA/PLA control specimen.

Figure 1 shows the 100 mm × 25 mm × 4 mm specimen models, which was constructed with computer-aided design (CAD) software. The PLA/TPU specimens were prepared using an Ultimaker 2+ 3D FDM machine by depositing a 2 mm thick TPU component on top of a 2 mm thick PLA component. The 3D printing filaments used for this study were commercial grade of PLA and TPU by Ultimaker. The material properties of the filaments were summarized in Table 1 [29,30].

The FDM deposition path was generated with a slicer software Cura 4.12 based on the FDM printing parameters, as shown in Table 2. The printing sequence was selected because TPU has a lower viscosity than PLA. Material with lower viscosity can easily infiltrate void spaces on the uneven surface of a higher-viscosity material, providing an additional contact area to improve the surface bonding strength between the two materials [31]. The printing parameters vary according to type of material, as they affect the quality of the print and adhesion. The printing speeds for the PLA and TPU layers were set differently according to the printing speed limit by the 3D printer and settings used by other researchers [10,17,18,32]. For this paper, the specimen was printed by considering the simplicity of the shape, dimension accuracy, and adhesion between materials of the specimen at speeds of 70 mm/s and 30 mm/s for PLA and TPU, respectively. The same printing speed settings for PLA and TPU as in Table 2 were applied by Brancewicz-Steinmetz et al. in their work to study the PLA/TPU multi-material interlayer bonding [17]. The build bed temperature was maintained at 60 °C throughout the FDM process of PLA and TPU based on the recommended bed temperature for PLA because it was meant to maintain adhesion between PLA and the build bed [32]. A PLA/PLA specimen, shown in Figure 1c, was prepared as the control specimen by printing a 2 mm PLA layer on top of a 2 mm PLA layer.

## 3. Experimental Methods

### 3.1. Thermoforming Test by Bending

**Figure 2:**
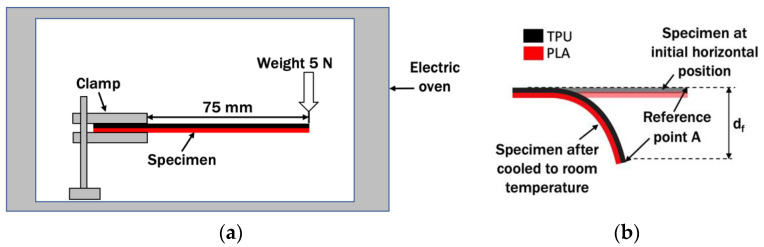
Bending test setup. (**a**) Specimen clamping and location of load; (**b**) measurement of vertical displacement of point A.

From the literature, no known standard was found to directly test the thermoformability characteristics of a multi-material layered plastic. In most cases, specific test methods such as bending and molding were set to address the needs of the products [4,24,27,28]. In order to evaluate the ease of the multi-material specimen to change shape under load and the thermoforming temperature for the purpose of this paper, a simple cantilever bending experiment was proposed. Specimens prepared in Section 2 complied with the ASTM D747-2 standard test method for the apparent bending modulus of plastics by means of a cantilever beam [33]. This standard was suited for determining relative flexibility of materials that are too flexible for test method ASTM D790. For this thermoforming test by bending, the PLA/TPU specimen was clamped horizontally as a cantilever with a 5 N load attached at the other end, as shown in Figure 2. This setup was proposed to simulate the typical thermoforming when the load is exerted at the end of the specimen to bend the specimen at the clamp point. The setup was then heated at a thermoforming temperature of 60 °C inside an electric oven for 5 min. Vise and weighting system were not used according to the ASTM D747-2 due to limited space in the electric oven. The specimen was removed from the oven and cooled at room temperature. The weight was then removed. The vertical displacement of the tip of the specimen at point A, d_f_, was measured, as shown in Figure 2b. The thermoforming test by bending was repeated with different thermoforming temperatures, T_f_, of 70 °C, 80 °C, 90 °C, 100 °C, and 110 °C. Tests for all conditions were repeated three times to obtain the averaged values of vertical displacement. With the same test procedure, a PLA/PLA control specimen was heated at 60 °C. The vertical displacement of the specimen was recorded and compared with those of PLA/TPU specimens.

In order to quantify the bending under different thermoforming temperatures, the apparent bending modulus (E) was approximated by Equation (1) based on the cantilever beam theory in ASTM D747-2 [33]:

E = 4 M S/(w b^3^ φ), (1)

where M is actual bending moment, S is the span length of the specimen, w is width of the specimen, b is the depth of the specimen, and φ is the bend angle measured in radians.

### 3.2. Thermoforming Test by Molding

**Figure 3:**
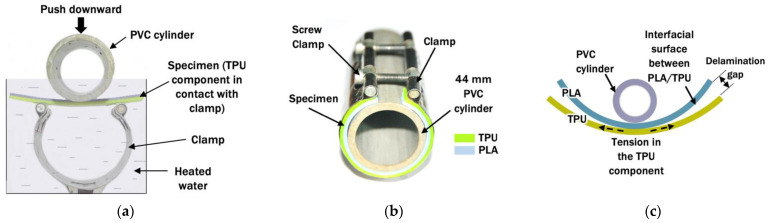
Thermoforming test by molding setup. (**a**) Molding of PLA/TPU specimen while submerged in heated water; (**b**) thermoformed specimen is secured with clamp and cooled at room temperature; (**c**) possible delamination along the interfacial surface of PLA/TPU specimen.

In the literature, the ends of male parts made of single material were pressed against a designer mold and their thermoformed shapes were discussed in molding tests [4,24]. This method was not suitable to evaluate the adhesion between the multi-material part, as it was designed for a single-material part. A thermoforming test by molding was proposed to investigate the interfacial surface bonding between PLA and TPU when being thermoformed to a circular shape at different temperatures. The PLA/TPU specimen was placed into temperature-controlled water for 5 min. The water was heated with an induction heater and its temperature was controlled at the desired thermoforming temperature with the heater’s built-in electronic controller. A digital thermometer was also used to ensure the water temperature was uniformly and correctly controlled. In this experiment, a similar specimen heating setup for a 3D-printed layered PLA/TPU shape memory experiment was adopted from Hasanzadeh et al. [25]. The specimen was then pressed into the clamp with a 44 mm diameter PVC cylinder, as shown in Figure 3a. The molding process was similar to the common three-point bending test for adhesion measurement. The TPU component, which is known to be more elastic than the PLA component, was assigned as the outer shell of the bending curve and subjected to tensile stress. It resulted in an opposite direction of the surface adhesion against the PLA component and possible delamination.

The clamp screws were tightened slowly until the PLA/TPU specimen and the PVC cylinder’s surface were fully in contact, as illustrated in Figure 3b. In order to maintain the thermoforming temperature, the specimen was kept fully submerged inside the heated water during the pressing and clamping process. Once finished, the specimen was cooled to room temperature before the clamp was removed. The shape thermoforming accuracy was determined by comparing the thermoformed specimen with the PVC cylinder used as a pattern in the molding process. Any delamination that occurred due to weak interfacial surface adhesion propagated along the interface and resulted in a gap, as shown in Figure 3c, to be visible and measured. The specimen tests were performed with different thermoforming temperatures, T_f_, of 60 °C, 70 °C, 80 °C, 90 °C, and 100 °C.

### 3.3. Shape Memory Test

**Figure 4:**
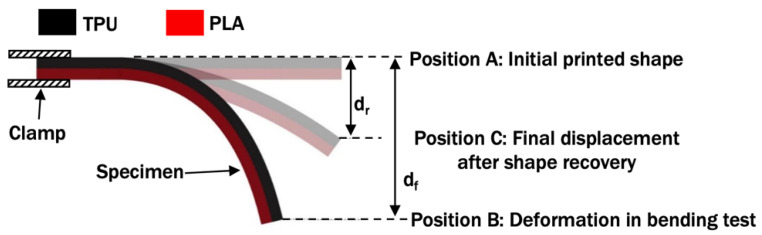
Shape recovery measurement of bent PLA/TPU specimen.

A shape memory test was carried out to investigate the ability of the deformed PLA/TPU specimens to return to their initial printed shape after being thermoformed at different temperatures. It is known that PLA and TPU alone possess some shape memory capacity. Various methods were presented by researchers, such as in [27,28]. However, there is no standard test to evaluate the shape memory ability of a thermoformed specimen or the size of the test specimen known to the author at this moment. Therefore, a shape memory test based on previous cantilever bending setup was applied in this paper. The specimens, which were bent in the previous bending, were heated for 5 min at a reheat temperature (T_r_) of 60 °C. This temperature is just above the PLA glass transition temperature and below the TPU heat deflection temperature. This temperature was used in a previous study by Jing et al. to investigate the shape memory of PLA/TPU [34]. The new vertical deformation of the specimens, d_r_, as shown in Figure 4, were measured. This test procedure was also conducted for the bent PLA control. The shape recovery percentage (SR) was determined by Equation (2) [28]:

SR = (d_f_ − d_r_)/d_f_ × 100%, (2)

where d_f_ and d_r_ are illustrated in Figure 4 as the vertical displacement of point A from position A to position B and the vertical displacement of point A from position A to position C, respectively. Tests for all conditions were repeated three times to ensure the repeatability of shape recovery of vertical displacement.

## 4. Results and Discussion

### 4.1. Influence of Thermoforming Temperature on Bending

**Figure 5:**
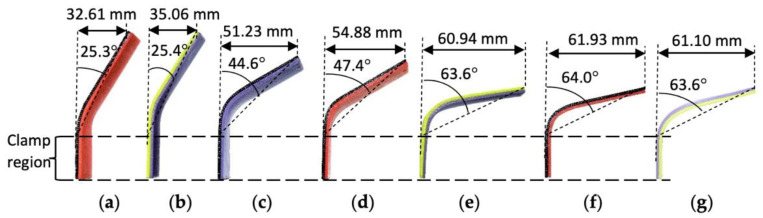
Comparison of PLA/TPU and PLA control specimens bent at different thermoforming temperatures: (**a**) T_f_ = 60 °C; (**b**) T_f_ = 70 °C; (**c**) T_f_ = 80 °C; (**d**) T_f_ = 90 °C; (**e**) T_f_ = 100 °C; (**f**) T_f_ = 110 °C; (**g**) PLA/PLA control specimen, T_f_ = 60 °C.

**Figure 6:**
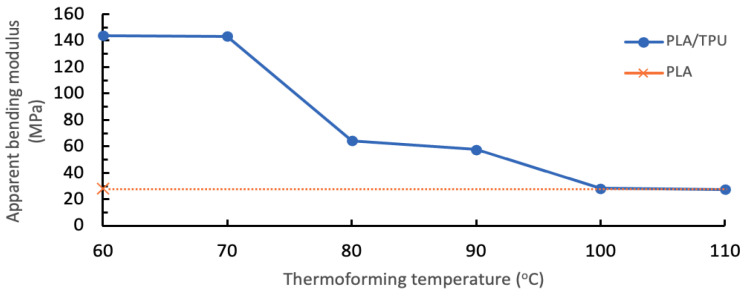
Apparent bending modulus for PLA/TPU specimens at different thermoforming temperatures and PLA/PLA control specimen at T_f_ = 60 °C.

Bending experiments were conducted to evaluate the ease of the multi-material specimen to change shape under load for different thermoforming temperatures, as shown in Figure 5. During the bending in an elevated temperature environment, the PLA/TPU cantilever specimens were observed to start deflecting downward when the temperature had reached near or above 60 °C. The deflection during the bending test became larger as the thermoforming temperature, T_f_, was increased, as recorded in Table 3. Each test condition was repeated three times. The results were consistent for all three repetitions, with the highest standard deviation of only 1.65 mm. The increment of deflections corresponded to the enlargement of the bend angle. The bend angles were applied in Equation (1) to approximate the apparent bending modulus, as shown in Figure 6.

For the temperatures of 60 °C and 70 °C, the thermoformed displacements and bend angle of the PLA/TPU specimen were almost identical. This temperature range is between the glass transition temperature (T_g,pla_) of PLA and the heat deflection temperature of TPU. Heat deflection temperature refers to the temperature at which a polymer deforms a specified distance under a load. A neat PLA has its heat deflection temperature at approximately 55 °C [35]. The PLA component loses its stiffness at temperatures above its glass transition temperature [36]. This was evidenced by the apparent bending modulus estimated at about 143 MPa. It is almost double the flexural modulus of TPU at 78.7 MPa, but less than 5% of the flexural modulus of PLA, as shown in Table 1 [29,30]. This results in softening of the PLA component, but it was supported by the TPU component of the specimen. At these lower thermoforming temperatures, the TPU was not deformed plastically as the PLA was. The elasticity of the TPU component countered the bending force and resisted the deflection. Furthermore, the TPU component was responsible for returning the deflection slightly when the bending load was removed. As the PLA component possessed a small degree of flexibility, it allowed the bonded TPU component to pull it back during the cooling process. This reduced the deformation inflicted by the thermoforming process. The modulus of PLA/TPU being higher than the flexural modulus of TPU can be attributed to the formation of a link between PLA and TPU at the interface. The cross-linking of polymer chains between PLA and TPU at the interface prevents the sliding of polymer chains and delays the glassy state transition to the rubbery state, thus improving its modulus [37].

The PLA component of the specimens, which was constantly being heated during printing, may undergo an annealing process due to re-crystallization of PLA. However, the effect of annealing for the PLA component due to bed heating during printing can be neglected because the exposure time is short. During the preparation of the specimen, precaution was taken such that only one specimen was printed at a time to maintain consistency of the print. The printing time of a specimen was less than 10 min. A neat PLA requires 20 to 40 min reach half-crystallization [38]. This finding is consistent with the results of the heat deflection temperature of PLA to remain at approximately 55 °C to 60 °C for the mold temperature of 60 °C [38]. This further supports the fact that the PLA component started to soften when the T_f_ was greater than its T_g,pla_.

The specimens were significantly deformed once the thermoforming temperatures were at 80 °C or higher. The TPU was no longer able to provide support because the TPU had been heated over its heat deflection temperature and lost its strength temporarily. The estimated apparent bending modulus for PLA/TPU specimens declined from 64.3 MPa at 80 °C to 27.6 MPa at 110 °C. These apparent bending moduli were lower than the flexural modulus of TPU. The drastic decline at 80 °C was because TPU started to lose its elasticity when heated over its heat deflection temperature. All the PLA/TPU specimens remained bonded through the tests. As the bend angle reached almost 90º, the deformation became constant at T_f_ = 100 °C and T_f_ = 110 °C due to the fact that it reached its limitation of the downward load.

As a comparison, the PLA/PLA control specimen was found to be bent to the maximum when a thermoforming temperature of 60 °C was applied. The control specimen retained its thermoformed shape even after the weight was removed, similar to those PLA/TPU specimens at the thermoforming temperature of 100 °C and above. The apparent bending modulus was much lower, at 28.2 MPa, compared to the PLA/TPU specimens of similar thermoforming temperatures. This clearly exhibited the role of the TPU component in preserving the bending strength of the PLA/TPU specimen at those thermoforming temperatures.

### 4.2. Influence of Thermoforming Temperature on Molding

**Figure 7:**
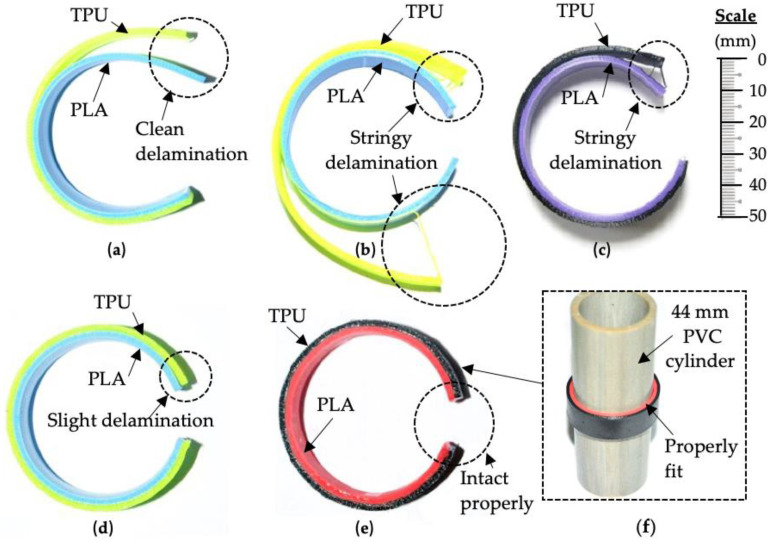
Top views of PLA/TPU specimens thermoformed into circular shape: (**a**) T_f_ = 60 °C; (**b**) T_f_ = 70 °C; (**c**) T_f_ = 80 °C; (**d**) T_f_ = 90 °C; (**e**) T_f_ = 100 °C; (**f**) shape accuracy of thermoformed specimen at T_f_ = 100 °C.

Figure 7 shows the multi-material specimens that were molded at different thermoforming temperatures and cooled at room temperature. It shows the shape of the specimens being released from the clamp and PVC pipe. The adhesion and shape retention accuracy after molding are observed and discussed. Figure 7a–d reveals that the adhesion at the interfacial surface of the PLA/TPU specimens failed during the molding at thermoforming temperatures between 60 °C and 90 °C. The delaminations between the TPU components from PLA components were observed immediately once the clamps were removed after the cooling process. This indicated that the delamination might have occurred during the molding or cooling process. During the molding process into circular shape, maximum shear occurred along the interfacial surface between the PLA and TPU components due to reactions that acted in the opposite directions. This was in line with the theory of mechanics of material related to a three-point bending of a beam such that the maximum shear stress, τ_max_, occurred at the neutral axis. After the cooling process, the TPU component restored most of its elastic properties. The elastic force of the TPU component increased as it was stretched farther along the PVC circular shape during the thermoforming test by molding. When being released from the mold, the TPU component tended to return to its original shape, while the thermoformed PLA component reacted to restrict the action of the TPU component. Due to these strong reactions, the interfacial surface bonding between the materials eventually failed and delamination between the two layers occurred.

In the thermoformed specimens at 60 °C, clean delamination was observed, as shown in Figure 7a. This indicated that complete delamination occurred at the interfacial surface of PLA/TPU. The shape of the PLA component, which was not exact to the circular shape of the PVC pipe, revealed that the PLA component still retained certain elasticity during the thermoforming process. This elasticity was supported by the finding in the bending experiment. Similar clean separation between PLA and TPU was observed during shear and tensile tests investigated by Brancewicz-Steinmetz et al. [17] and Tamburrino et al. [39], respectively. In their studies, the adhesion between the PLA/TPU interface was concluded as weak because it only sustained a shear strength of 0.26 MPa and tensile stress of 0.28–0.44 MPa. This further justified the clean delamination observed for the thermoformed specimen at 60 °C.

The thermoformed PLA/TPU specimen at a temperature of 70 °C in Figure 7b showed the worst delamination propagated along the interfacial surface. The TPU component still maintained its elastic properties, as the thermoforming temperature was below the TPU’s heat deflection temperature. However, its elastic action was restricted by the thermoformed PLA component at 70 °C, making it worse than the PLA/TPU specimen at 60 °C. The restricting action of the thermoformed PLA component was clearly shown by its shape accuracy. The shape accuracy of the thermoformed PLA component for temperatures over 70 °C shown in Figure 7b–e greatly improved and maintained to the circular shape of the PVC pipe. Due to the strong elastic action of the TPU component, delamination happened when it exceeded the adhesion between the interfacial surface. Only a few stringy delaminations from the TPU component were observed for specimens being thermoformed at 70 °C and 80 °C. This showed that the bonding within the TPU component might be weaker near the heat deflection temperature than those between the interfacial surface and resulted in delamination within the TPU components. When the thermoforming temperature reached or exceeded the TPU’s heat deflection temperature, the TPU components underwent plastic deformation and subsequently lost their elasticity. These occurrences were observed with a small delamination for specimens at T_f_ of 80 °C and 90 °C.

No delamination was seen from thermoforming results at T_f_ = 100 °C. This specimen at T_f_ = 100 °C was perfectly fit to the circular shape of the PVC cylinder and the PLA/TPU interfacial surface bonding was intact, as shown in Figure 7f. This indicated that both PLA and TPU components fully deformed plastically and were supported by their large bend angle seen in the results of the bending tests. The high thermoforming temperature at T_f_ = 100 °C may promote the lamination bond to be re-established during the molding process, as it is closer to the hot lamination temperature of 180 °C used by Ji et al. to laminate two flat PLA and TPU together [13].

In fact, previous study by Rosa et al. concluded that the bonding of the PLA/TPU surface is limited and only lasts for few load cycles [40]. Brancewicz-Steinmetz et al. stated that the interfacial surface bonding of PLA/TPU is considerably weak and relies mostly on the surface roughness of the PLA [17]. These factors further contributed to the delamination of PLA/TPU specimens when thermoforming temperatures are not selected properly.

### 4.3. Shape Recovery of Thermoformed Specimens

**Figure 8:**
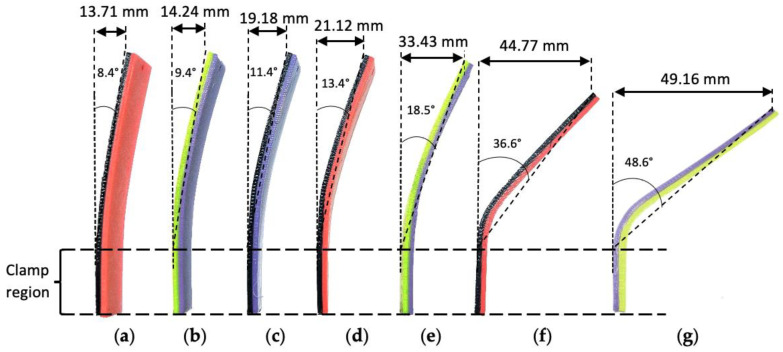
Shape after recovery for PLA/TPU specimens and PLA control specimens that had been bent at different thermoforming temperatures: (**a**) T_f_ = 60 °C; (**b**) T_f_ = 70 °C; (**c**) T_f_ = 80 °C; (**d**) T_f_ = 90 °C; (**e**) T_f_ = 100 °C; (**f**) T_f_ = 110 °C; (**g**) PLA/PLA control specimen at T_f_ = 60 °C.

A shape recovery test of thermoformed specimens was performed through heat stimulus of 60 °C. Figure 8 shows the shape of the thermoformed PLA/TPU specimens after being exposed to heat stimulus. All thermoformed specimens showed an ability to slightly recover to the previous shape after being heated at T_r_ = 60 °C. The bend angles of the PLA/TPU specimens in Figure 8 were measured. None of the specimens were able to fully restore their original shape, as expected. This was because the pre-deformed PLA and TPU only recorded a shape recovery percentage of 9% and 87% under a heat stimulus of 60 °C, respectively [41]. No delamination was observed for any specimen after shape recovery.

**Figure 9:**
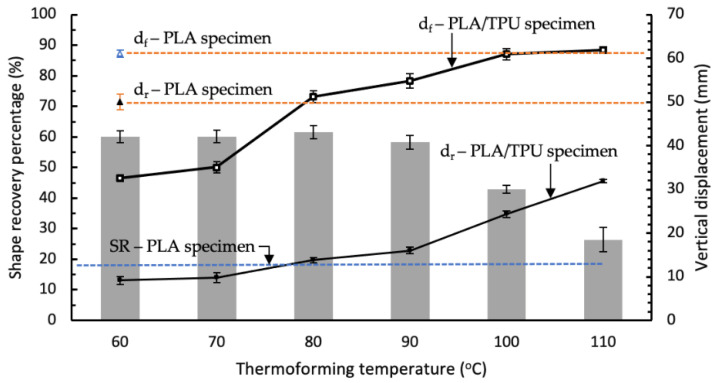
The relationships of shape recovery ability of specimens with thermoforming temperatures.

The shape recovery was quantified by comparing the change in bend angle of the PLA/TPU specimens before and after being exposed to heat stimulus. The percentages of the shape recovery for PLA/TPU specimens that were thermoformed at different temperatures were estimated using Equation (2) and are summarized in the bar chart in Figure 9. A heat stimulus of 60 °C was selected based on the glass transition temperature for PLA as the common switching temperature for shape memory polymer [42]. From Figure 9, it can be seen that the percentages of the shape recovery for PLA/TPU specimens being thermoformed at T_f_ = 60 °C to 90 °C were estimated to be almost similar in the range of 58.3% to 61.6%. The highest shape recovery percentage occurred for specimens being thermoformed at T_f_ = 80 °C. This agrees with findings in the literature. Ehrmann et al. suggested that the recovery temperature should be increased to at least 70 °C to enable a higher recovery rate of 3D-printed PLA structures [43]. The shape recovery rate increment was observed when the temperature was increased in the range of 50 °C to 80 °C for the 80/20 TPU/PLA blend specimen in the stretch shape recovery test conducted by Song et al. [44]. Lai et al. reported a lower shape recovery ratio of 32% for their 50/50 PLA/TPU melt-blended specimen that was pre-deformed at 80 °C and underwent recovery at 60 °C [41]. This can be attributed to the better collective recovery action of the TPU component of PLA/TPU specimen in this paper than those in the melt-blended specimen.

However, this shape recovery ability rapidly declined for the specimens being thermoformed at higher thermoforming temperatures of 100 °C and 110 °C, as shown in Figure 9. This trend is consistent with the finding by Wu et al., such that the higher deformation temperature results in a lower shape recovery ratio of the PLA specimens [45]. Lai et al. reported a significantly low shape recovery ratio of about 8% for their 50/50 PLA/TPU melt-blended specimen that was pre-deformed at 120 °C and underwent recovery at 60 °C [41]. The shape recovery percentage for the specimen bent at T_f_ = 110 °C approached the result of the PLA control specimen. This result showed that the shape memory ability of the PLA/TPU specimens was mainly contributed by the elasticity of the TPU layer. Once the TPU layer was thermoformed above its heat deflection temperature and lost its elasticity, the shape memory ability of the specimens decreased accordingly.

## 5. Conclusions

Thermoforming of a multi-material PLA/TPU part fabricated using the FDM process was investigated and found to be a suitable method for rapid prototyping. This study identified the elasticity of the TPU component as the key element in the characteristics of the multi-material PLA/TPU thermoforming. These characteristics changed when the thermoforming temperature was altered. The following conclusions were drawn from this study:

- Simple thermoforming and shape memory tests were introduced for an FDM-fabricated specimen. From the test results, the characteristics of multi-material specimens were quantified using apparent bending modulus and shape recovery percentage. In addition, adhesion between the interface and the mold accuracy of the multi-material specimen undergoing thermoforming into circular shape can be observed.
- The PLA/TPU specimens being thermoformed at T_f_ = 60 °C to 70 °C exhibited a similar apparent bending modulus of 143 MPa, which was higher than the TPU’s flexural modulus of 78.7 MPa. This was attributed to the bonding of PLA and TPU at the interface that prevented sliding of the polymer chain and delayed the glass transition to the rubbery state. The apparent bending modulus of PLA/TPU declined drastically from 64.3 MPa at T_f_ = 80 °C to 27.6 MPa at T_f_ = 110 °C due to weakening of the elasticity of TPU after reaching its heat deflection temperature.
- The thermoforming test by molding into a circular shape provided insights related to adhesion between the interface of PLA/TPU specimens. Adhesion improved as the thermoforming temperature increased. This can be attributed to the exposure time and heat for the bonding to be formed via re-crystallization. Specimens at T_f_ = 100 °C and T_f_ = 110 °C showed good shape retention accuracy and interfacial surface bonding after the thermoforming process.
- PLA/TPU specimens being thermoformed at T_f_ = 60 °C to 90 °C showed reasonable shape recovery with their shape memory percentage in the range of 58.3% to 61.6%. The shape memory dropped rapidly below 50% when being thermoformed at T_f_ greater than 100 °C. These findings were consistent with the literature regarding PLA and TPU/PLA blend specimens. The test results indicated that shape accuracy and the shape memory ability of the thermoformed prints did not coexist.
- With the knowledge of shape recovery and the molding process by the PLA/TPU specimen, the proper thermoforming temperature is suggested to be at 100 °C to 110 °C for applications where permanent parts are desired. However, when the parts should allow slight shape recovery such as in splints and casts for medical purposes, a thermoforming temperature of 90 °C to 100 °C is recommended. Parts are suggested to be thermoformed at a temperature between 80 °C to 90 °C if the parts are subjected to bending load only. These suggested thermoforming temperatures have taken delamination issue and part accuracy into consideration. This enables the faster development of products with different rigidity compositions and predetermined shape memory abilities.

The adhesion strength between PLA and TPU printed directly from an FDM process remains a major limitation for its potential in thermoforming accuracy and shape recovery. Future studies will focus on the interlayer geometry of the thermoforming process and the effects of different compositions of TPU on PLA/TPU thermoforming.

## Figures and Tables

**Table 1:** Mechanical properties of PLA and TPU filament [29,30].

Properties	PLA	TPU
Diameter (mm)	2.85 ± 0.10	2.85 ± 0.10
Tensile modulus (MPa)	2346.5	26.0
Tensile stress at yield (MPa)	49.5	8.6
Tensile stress at break (MPa)	45.6	39.0
Elongation at yield (%)	3.3	55.0
Elongation at break (%)	5.2	580.0
Flexural strength (MPa)	103.0	4.3
Flexural modulus (MPa)	3150.0	78.7
Melt mass-flow rate (g/10 min)	6.1	15.9
Glass transition (°C)	~60.0	−24.0
Heat deflection at 0.455 MPa (°C)	-	74.0

**Table 2:** FDM printing parameters for different components [10,17,18].

Printing Parameter	PLA Component	TPU Component
Layer height (mm)	0.15	0.15
Nozzle diameter (mm)	0.4	0.4
Infill (%)	90	90
Wall count	3	3
Top/Bottom layers	3	3
Nozzle temperature (°C)	210	220
Build bed temperature (°C)	60	60 *
Printing speed (mm/s)	70	30

* Set based on recommended temperature for PLA to maintain adhesion of PLA to build bed.

**Table 3:** Averaged vertical displacement of specimens under bending test.

T_f_ (°C)		60	70	80	90	100	110
d_f_ (mm)	PLA/TPU	32.61 ± 1.21	35.06 ± 1.65	51.23 ± 0.95	54.88 ± 1.04	60.94 ± 1.07	61.93 ± 0.56
	PLA/PLA	61.10 ± 0.87	-	-	-	-	-

## Data Availability

Not applicable.

## References

[1] Chua B.-L., Baek S.-H., Park K., Ahn D.-G. (2021). Numerical Investigation of Deposition Characteristics of PLA on an ABS Plate Using a Material Extrusion Process. Materials.

[2] Vyavahare S., Teraiya S., Panghal D., Kumar S. (2020). Fused Deposition Modelling: A Review. RPJ.

[3] Fitzpatrick A.P., Mohanned M.I., Collins P.K., Gibson I. (2017). Design of a Patient Specific, 3D Printed Arm Cast. KEG.

[4] Mustakangas A., Iso-Junno T., Jokelainen T., Keskitalo M., Mäntyjärvi K. (2019). Forming and Formability of 3D Printed Thermoplastics. AIP Conf. Proc..

[5] Mäntyjärvi K., Iso-Junno T., Mustakangas A., Jokelainen T., Keskitalo M., Järvenpää A. (2019). Exploitation of Forming of the 3D Printed Materials. AIP Conf. Proc..

[6] Nkomo N.Z., Gwamuri J., Sibanda N.R., Nkiwane L.C. (2017). A Study of Applications of 3D Printing Technology and Potential Applications in the Plastic Thermoforming Industry. IOSR J. Eng..

[7] Ilyas R.A., Sapuan S.M., Harussani M.M., Hakimi M.Y.A.Y., Haziq M.Z.M., Atikah M.S.N., Asyraf M.R.M., Ishak M.R., Razman M.R., Nurazzi N.M. (2021). Polylactic Acid (PLA) Biocomposite: Processing, Additive Manufacturing and Advanced Applications. Polymers.

[8] Owen J. Enabling R&D—Thermo-Forming Trials. https://enablingthefuture.org/2015/05/04/enabling-rd-thermo-forming-trials/.

[9] Popescu D., Zapciu A., Tarba C., Laptoiu D. (2020). Fast Production of Customized Three-Dimensional-Printed Hand Splints. RPJ.

[10] Sorimpuk N.P., Choong W.H., Chua B.L. (2022). Design of Thermoformable Three Dimensional-Printed PLA Cast for Fractured Wrist. IOP Conf. Ser. Mater. Sci. Eng..

[11] Choi W., Jang W., Kim J., Kim J.-H., Hwang S. A Pilot Study for Usefulness of Customized Wrist Splint by Thermoforming Manufacturing Process Using 3D Printing: Focusing on Comparative Study with 3D Scanning Manufacturing Process. Proceedings of the Resna Annual Conference.

[12] Razzaq M.Y., Gonzalez-Gutierrez J., Mertz G., Ruch D., Schmidt D.F., Westermann S. (2022). 4D Printing of Multicomponent Shape-Memory Polymer Formulations. Appl. Sci..

[13] Ji X., Gao F., Geng Z., Li D. (2021). Fabrication of Thermoplastic Polyurethane/Polylactide Shape-Memory Blends with Tunable Optical and Mechanical Properties via a Bilayer Structure Design. Polym. Test..

[14] Liu X., Zhou L., Heng P., Xiao J., Lv J., Zhang Q., Hickey M.E., Tu Q., Wang J. (2019). Lecithin Doped Electrospun Poly(Lactic Acid)-Thermoplastic Polyurethane Fibers for Hepatocyte Viability Improvement. Colloids Surf. B Biointerfaces.

[15] Tao Y., Shao J., Li P., Shi S.Q. (2019). Application of a Thermoplastic Polyurethane/Polylactic Acid Composite Filament for 3D-Printed Personalized Orthosis. Mater. Tehnol..

[16] Dogan S.K., Boyacioglu S., Kodal M., Gokce O., Ozkoc G. (2017). Thermally Induced Shape Memory Behavior, Enzymatic Degradation and Biocompatibility of PLA/TPU Blends: “Effects of Compatibilization. ” J. Mech. Behav. Biomed. Mater..

[17] Brancewicz-Steinmetz E., Sawicki J., Byczkowska P. (2021). The Influence of 3D Printing Parameters on Adhesion between Polylactic Acid (PLA) and Thermoplastic Polyurethane (TPU). Materials.

[18] Kepenekci M. (2021). Mechanical Behavior of Additively Manufactured Polymer Composite Structures and Interfaces.

[19] Zhang X. (2021). 3D Printing of Bio-Inspired, Multimaterial Structures to Enhance Stiffness and Toughness.

[20] An B., Tao Y., Gu J., Cheng T., Chen X., Zhang X., Zhao W., Do Y., Takahashi S., Wu H.-Y. Thermorph: Democratizing 4D Printing of Self-Folding Materials and Interfaces. Proceedings of the 2018 CHI Conference on Human Factors in Computing Systems.

[21] Kačergis L., Mitkus R., Sinapius M. (2019). Influence of Fused Deposition Modeling Process Parameters on the Transformation of 4D Printed Morphing Structures. Smart Mater. Struct..

[22] García Plaza E., Núñez López P., Caminero Torija M., Chacón Muñoz J. (2019). Analysis of PLA Geometric Properties Processed by FFF Additive Manufacturing: Effects of Process Parameters and Plate-Extruder Precision Motion. Polymers.

[23] Dizon J.R.C., Espera A.H., Chen Q., Advincula R.C. (2018). Mechanical Characterization of 3D-Printed Polymers. Addit. Manuf..

[24] Ekşi O. (2020). Plug Production for Thermoforming Using Fused Deposition Modelling. J. Polytech..

[25] Hasanzadeh A., Golzar M. (2020). 3D Printing of Shape-Memory Polymer Based on Polylactic-Acid and Thermoplastic-Elastomer: Investigating of Shape-Memory and Thermo-Viscoelastic Properties. Modares Mech. Eng..

[26] Rossing L., Scharff R.B.N., Chömpff B., Wang C.C.L., Doubrovski E.L. (2020). Bonding between Silicones and Thermoplastics Using 3D Printed Mechanical Interlocking. Mater. Des..

[27] Wang Y., Li X. (2021). 4D-Printed Bi-Material Composite Laminate for Manufacturing Reversible Shape-Change Structures. Compos. Part B Eng..

[28] Li F., Scarpa F., Lan X., Liu L., Liu Y., Leng J. (2019). Bending Shape Recovery of Unidirectional Carbon Fiber Reinforced Epoxy-Based Shape Memory Polymer Composites. Compos. Part A Appl. Sci. Manuf..

[29] Ultimaker Ultimaker PLA Technical Data Sheet V3.011—Korean. https://support.ultimaker.com/hc/en-us/article_attachments/360010199919/TDS_PLA_v3.011-kor.pdf.

[30] Ultimaker Ultimaker TPU 95A Technical Data Sheet V3.010—Korean. https://support.ultimaker.com/hc/en-us/article_attachments/360010207099/TDS_TPU_95A_v3.010_KOR.pdf.

[31] Harris C.G., Jursik N.J.S., Rochefort W.E., Walker T.W. (2019). Additive Manufacturing With Soft TPU—Adhesion Strength in Multimaterial Flexible Joints. Front. Mech. Eng..

[32] Ultimaker Ultimaker 2+ User Manuals. https://support.ultimaker.com/hc/en-us/articles/360011811480-Ultimaker-2-user-manuals.

[33] (2010). Standard Test Method for Apparent Bending Modulus of Plastics by Means of a Cantilever Beam.

[34] Jing X., Mi H.-Y., Peng X.-F., Turng L.-S. (2015). The Morphology, Properties, and Shape Memory Behavior of Polylactic Acid/Thermoplastic Polyurethane Blends. Polym. Eng. Sci..

[35] Tábi T., Ageyeva T., Kovács J.G. (2021). Improving the Ductility and Heat Deflection Temperature of Injection Molded Poly(Lactic Acid) Products: A Comprehensive Review. Polym. Test..

[36] Péter T., Litauszki K., Kmetty Á. (2021). Improving the Heat Deflection Temperature of Poly (Lactic Acid) Foams by Annealing. Polym. Degrad. Stab..

[37] Lee C.H., Khalina A., Lee S.H. (2021). Importance of Interfacial Adhesion Condition on Characterization of Plant-Fiber-Reinforced Polymer Composites: A Review. Polymers.

[38] Tábi T., Ageyeva T., Kovács J.G. (2022). The Influence of Nucleating Agents, Plasticizers, and Molding Conditions on the Properties of Injection Molded PLA Products. Mater. Today Commun..

[39] Tamburrino F., Graziosi S., Bordegoni M. (2019). The Influence of Slicing Parameters on the Multi-Material Adhesion Mechanisms of FDM Printed Parts: An Exploratory Study. Virtual Phys. Prototyp..

[40] Rosa F., Bordegoni M., Dentelli A., Sanzone A., Sotgiu A. (2017). Print-in-Place of Interconnected Deformable and Rigid Parts of Articulated Systems. Procedia Manuf..

[41] Lai S.-M., Lan Y.-C. (2013). Shape Memory Properties of Melt-Blended Polylactic Acid (PLA)/Thermoplastic Polyurethane (TPU) Bio-Based Blends. J. Polym. Res..

[42] Senatov F.S., Niaza K.V., Zadorozhnyy M.Y., Maksimkin A.V., Kaloshkin S.D., Estrin Y.Z. (2016). Mechanical Properties and Shape Memory Effect of 3D-Printed PLA-Based Porous Scaffolds. J. Mech. Behav. Biomed. Mater..

[43] Ehrmann G., Ehrmann A. Shape-Memory Properties of 3D Printed PLA Structures. Proceedings of the First International Conference on “Green” Polymer Materials.

[44] Song J.J., Srivastava I., Kowalski J., Naguib H.E. Fabrication and Characterization of a Foamed Polylactic Acid (PLA)/Thermoplastic Polyurethane (TPU) Shape Memory Polymer (SMP) Blend for Biomedical and Clinical Applications. Proceedings of the SPIE.

[45] Wu W., Ye W., Wu Z., Geng P., Wang Y., Zhao J. (2017). Influence of Layer Thickness, Raster Angle, Deformation Temperature and Recovery Temperature on the Shape-Memory Effect of 3D-Printed Polylactic Acid Samples. Materials.

